# Oral cryotherapy for management of chemotherapy‐induced oral mucositis in haematopoietic cell transplantation: a systematic review

**DOI:** 10.1186/s12885-022-09539-8

**Published:** 2022-04-22

**Authors:** Faizah Jabr Alsulami, Sadr ul Shaheed

**Affiliations:** 1 Susan Wakil School of Nursing and Midwifery, University of Sydney, Sydney, Australia; 2 Nuffield Department of Surgical Sciences and Biomedical Research Centre, University of Oxford, Oxford, UK

**Keywords:** Haematological malignancies, Chemotherapy, Oral cryotherapy, Oral mucositis, Haematopoietic cell transplantation

## Abstract

**Background:**

Oral mucositis (OM) is known to be the most common and challenging side effect of conditioning chemotherapy in haematopoietic cell transplant (HCT). This side effect causes significant morbidity and may delay the treatment plan, as well as increase therapeutic expenses. There are few clinical trials in the literature that indicate any kind of treatment or prevention methods are effective. Therefore, the aim of this study is to perform a systematic review of literature and examine the effectiveness of oral cryotherapy (OC) in management of chemotherapy-induced OM in patients with haematological malignancies undergoing a HCT.

**Methods:**

A systematic literature search was conducted, using the electronic databases PubMed, Embase, MEDLINE and Scopus. A total of 322 papers were identified and 9 papers were analysed based on defined inclusion and exclusion criteria. The quality of the chosen primary studies was appraised using the COCHRANE risk of bias assessment tool.

**Results:**

Nine randomized controlled trials, analysing 658 participants; control group (*n* = 289, age mean ± SD; 41.15 ± 21) and treatment group (*n* = 369, age mean ± SD; 39.15 ± 20), were included in this systematic review. Seven studies had significantly addressed the effectiveness of OC (*p* value < 0.05), in reducing the incidence of developing severe OM in the adult population undergoing HCT, especially when the conditioning regimen protocols included high dose of alkylating agent such as melphalan.

**Conclusion:**

This review supports the use of OC for prevention of OM in patients undergoing HCT, with high-dose of melphalan conditioning protocols. It is recommended that more studies be conducted to compare efficacy and duration of OC with other chemotherapeutic agents with relatively short plasma half-lives. The heterogeneity of the trials demonstrated the need to regulate the validated assessment tools and similar interventions that would enable comparisons and analyses of treatment effects based on well-designed RCTs.

## Background

Despite the development of anticancer drugs and therapies, the haematopoietic cell transplant (HCT) is an effective and curative treatment for specific types of blood cancer that affect the bone marrow, such as leukaemia, lymphoma and myeloma [1]. The number of HCT procedures has significantly increased over the last two decades, around 1.5 million transplants in more than 1,500 transplantation centres worldwide and 4,500 transplants in the USA in 2018 alone [2]. In Europe and collaborating countries, the number of HCT continues to rise to 48,512 in 43,581 patients, reported by 700 centres in 51 countries during 2019 [3]. A recent study by Nishimura et al. 2020 [4] investigated 4,329 multiple myeloma (MM) patients (median age 59), and found that patients receiving autologous stem cell transplantation after 2014 had a 23% increase in survival for five years or longer compared to those treated in 1997 or earlier. The number of registered donors of stem cells and cord blood units with the Bone Marrow Donor Association has been increasing, reaching approximately 25 million [5], which in turn has increased the number of procedures for cancer patients and enhanced their survival rate.

Chemotherapy infusions are used as an essential part of the HCT procedure to achieve two important goals: reduce the tumour burden and provide sufficient immunosuppression to prevent developing graft rejection after transplantation [6]. Traditionally, this target is achieved by receiving supralethal doses of total body irradiation (TBI) and chemotherapeutic agents with non-overlapping toxicities [7]. The conditioning regimens mostly consists of alkylating antineoplastic agents like melphalan, cyclophosphamide, busulfan, carmustine, or topoisomerase inhibitors like etoposide, based on its immunomodulatory properties [7]. In fact, higher doses of conditioning regimens could lead to serious complications such as fatal pulmonary, gastrointestinal and hepatic toxicities, as well as impaired growth and development in children [7, 8].

One of the most common and challenging side effects of conditioning chemotherapy is oral mucositis (OM). Mucositis is characterised by inflammation and/or ulcerative lesions located on the oral and/or gastrointestinal tract [9]. The incidence of chemotherapy-induced OM is up to 80% among patients receiving high-dose chemotherapy treatments [8]. Therefore, to accomplish early detection and prevention of OM associated with life-threatening complications, such as sepsis, which can lead to death, patients must undergo a comprehensive oral examination, during and after the completion of a chemotherapy course [9, 10]. Other complications that cancer patients may encounter due to OM are an increased consumption of narcotics to manage pain associated with mouth inflammation, an increased length of hospital stay and the inability to eat or drink, leading to the use of total parenteral nutrition (TPN) support [11]. These factors play a significant role in terms of increasing the cost of treatment and exposing patients to emotional and social distress (e.g. self-isolation, anxiety and depression) [12].

A number of agents and methods have been introduced as early preventive approaches to OM during HCT, including routine oral care, natural interventions such as oral cryotherapy, low-level laser therapy (LLLT), keratinocyte growth factor, methylene blue, melatonin, honey, mucosal protective agents, and antimicrobial agents [13–16]. Therefore, it is difficult to construct a well-designed, adequately-powered, and carefully-conducted randomised controlled trial to compare competing interventions because the current literature is insufficient to provide adequate assessment of the comparative efficacies of pharmacological and non-pharmacological therapies.

Oral cryotherapy (OC) involves cooling the patient’s mouth during chemotherapy infusion using ice cubes, cold water, popsicles, or ice cream to reduce the risk of developing chemotherapy-induced OM [17]. This intervention plays an important role in terms of decreasing blood circulation to the mouth by narrowing the blood vessels and thus reducing the amount of the chemotherapy drugs that is penetrating the mucous membrane [9]. OC also improves oral tissue preservation and reduces the metabolic function of epithelial and basal cells by significantly increasing the expression proinflammatory cytokines [18–20]. On other side cold neuralgia or sphenopalatine ganglioneuralgia caused by OC, may increase the delivery of bone-marrow stem cells to the human brain [21]. OC has been shown to effectively reduce the incidence and severity of oral mucositis in adult patients, receiving 5FU-based treatment for solid cancers [22, 23]. There is also evidence showing that OC can reduce the incidence and severity of OM in adults, after receiving high-dose melphalan-based chemotherapy prior to HCT [22, 23].

The efficacy of OC for patients receiving HCT with haematological malignancies has not been explored. Therefore, this systematic review aims to find out whether OC is effective in preventing severe OM and its influence on the onset or evolution of pain in patients receiving HCT with haematological malignancies. Thus, the appropriate treatment option can be provided for the management of chemotherapy induced oral mucositis in patients undergoing HCT.

## Methods

### Search strategy

To identify studies investigating the effectiveness of OC in preventing or reducing the occurrence of chemotherapy-induced OM among the mentioned population, a systematic literature search was conducted using the electronic databases; PubMed, Scopus, Embase and MEDLINE via OvidSP (1946–present), between 3 September and 20 December 2020. These databases were selected for their large number of publications related to the medical and health fields. For this systematic review, three primary search terms were used to perform the search syntax: chemotherapy-induced oral mucositis, haematopoietic stem cell transplantation and haematological malignancies. These terms were combined with the intervention oral cryotherapy using the search function ‘AND’. Variations, including synonyms for these terms, were also searched in combination with their primary concepts. This was conducted using the search function ‘OR’, after which these terms and their respective variations were combined using the search function ‘AND’.

To explore the topic as widely as possible, keywords comprising each concept were utilised to search without limiting it to subject headings. Additionally, different spellings and word forms were considered by truncating the search term and using an asterisk when applicable. No additional limits were applied in terms of date of publication, study design and language. Therefore, all records were screened manually by the two independent reviewers, checking the title and abstract as well as reading the full text when required.

### Inclusion criteria

- Human trials reporting on populations with haematological malignancies undergoing any type of HCT (either an allogeneic transplant or an autologous transplant) and receiving any type of high-intensity conditioning chemotherapy protocol that was associated with the risk of developing OM.
- Trials that clearly defined the type and duration of OC.
- Studies focussing on the impact of OC in terms of preventing OM.
- Studies that utilised a recognised scoring system for the assessment of OM severity to report patients’ outcomes after using interventions (e.g. the WHO’s grading of mucositis)
- Studies in the English language
- Peer-reviewed studies

### Exclusion criteria

- Books
- Conference and poster presentation
- Non-full-text articles
- Unrelated language
- Studies conducted on patients with a solid tumour (e.g. head and neck cancer)
- Studies using mixed interventions (e.g. a combination of OC and laser therapy to prevent chemotherapy-induced OM).

### Critical appraisal

The quality of the chosen primary studies was appraised using the COCHRANE risk of bias assessment tool [24]. Due to the paucity of primary studies on the topic, none of the included trials in this review was excluded based on poor methodology.

## Results

**Fig. 1:**
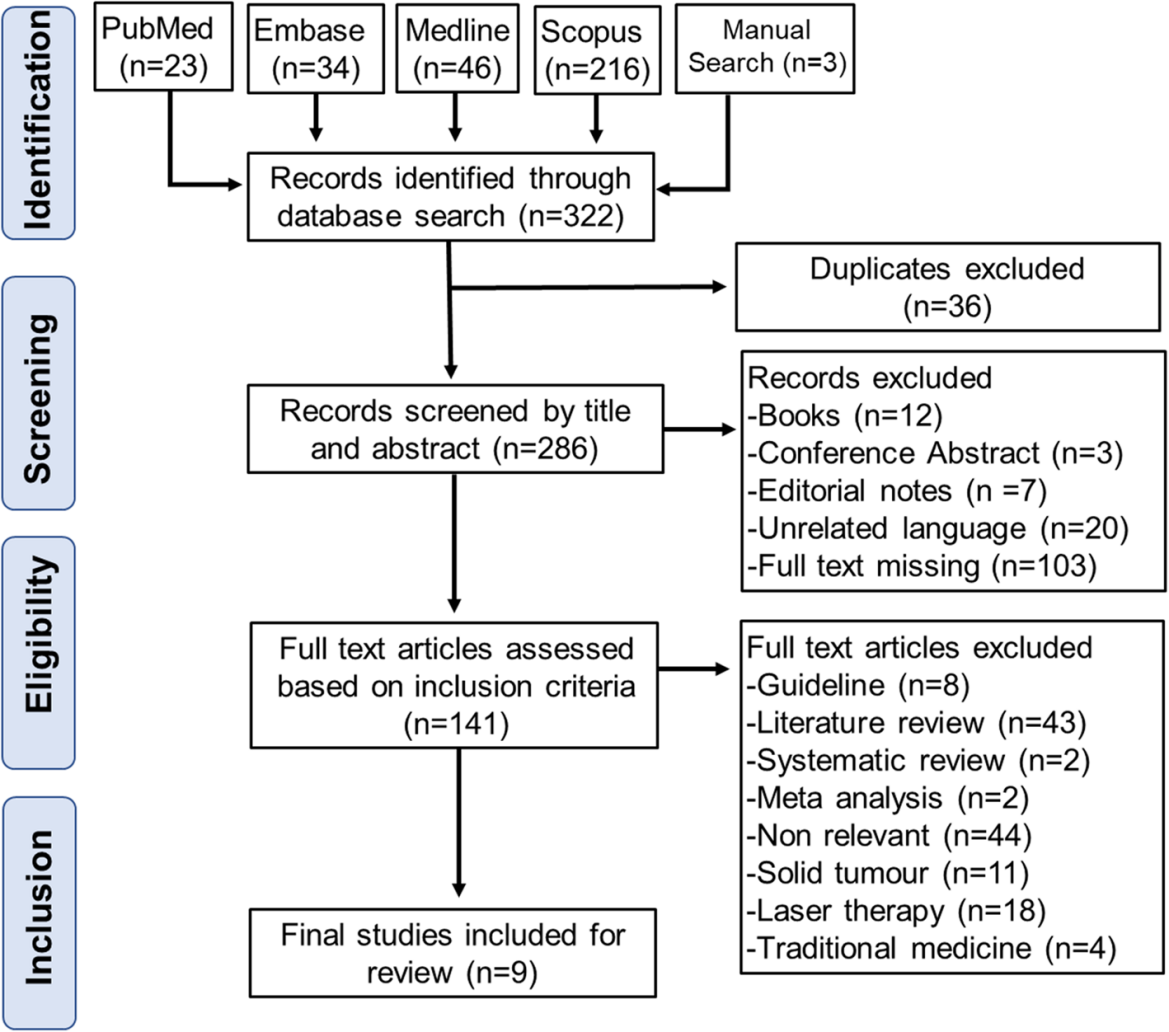
PRISMA workflow of the identification, screening, eligibility, and inclusion of the studies in the systematic review

The initial search outcomes included 322 records, with 3 records found through manual searching in references. The screening process was conducted on 286 records after removing duplicates. A total of 145 were removed following the primary screening of the titles and abstracts due to the following reasons: they were book publications, conference abstracts or editorial notes, had unrelated language and/or full text was missing through libraries and other sources. In the stage of full-text manual screening, a total of 141 articles were assessed against the inclusion criteria of the research question. As a result, 132 records were excluded for various reasons (Fig. 1). Thus, this review included nine primary studies as summarised in Tables 1 and 2. The studies were organised by dates, from oldest to most recent. This was done to allow for logical sequence of arrangement in terms of observing how the OC technique was improving through these years. Out of the 9 randomized control trials (RCTs), 7 studies had only addressed the impact of OC on preventing OM among adults [25–31]. Three studies in adult populations and one study in paediatric population had addressed the effect OC on preventing OM as well as measuring how this intervention had influenced the application of pain medications, nutritional status and duration of hospital stay [25, 26, 28, 32].

**Table 1:** Characteristics of included studies

Author and Year of Publication	Country, Time Frame, and Study Design,	Sample Size, Age, and Gender			Patient Diagnosis	Type of HCT	Type of Chemotherapy Regimen	Duration of Oral Cryotherapy treatment	Comparator
Lilleby et al. 2006 [25]	USA, 2003–2005, RCT	Age, median (range)	**Treatment**	**Control**	Myeloma	auto-HCT	Melphalan	To place one ounce of crushed ice into the mouth for 30 min before starting chemotherapy treatment and continue with the procedure for 6 h following the completion of infusion	Saline rinse
			59 (51–71)	57 (33–72)					
		Male	16	12					
		Female	5	7					
Gori et al. 2007 [33]	Italy, 2004–2006, Multicentre RCT	Age, median (range)	35.5 (9–59)	40 (8–66)	ALL, AML, MM CML, AA, CLL, Myelodysplasia, Thalassemia, Non-HL, Myelofibrosis,	allo-HCT,	Methotrexate-containing GvHD prophylaxis	OC applied for 60 min during infusion	No treatment
		Male	32	30					
		Female	30	30					
Svanberg et al. 2007 [26], Svanberg et al. 2010 [27]	Sweden, 2002–2004 RCT	Age, mean SD	49.8 14.4	54.3 11.0	MM, AML, ALL, LYMPHOMA, TC,	auto-HCT, allo-HCT	Melphalan BEAC, BEAM, Others	To start sucking ice chips or rinsing with ice cold water, concurrently with the chemotherapy infusion, and to continue until the end of session	Basic oral care
		Male	26	19					
		Female	13	20					
Salvador et al. 2012 [28]	Canada, 8 months, RCT	Age, mean SD	56.0 8.9	62.0 7.7	MM	auto-HCT	Melphalan	OC plus oral care (sodium bicarbonate mouthwash and gentle brushing for the teeth using sponges), In addition to the standard oral care, the patients sucked ice chips for a total of 60 min. To start procedure 5 min before infusion	Usual oral care
		Male	14	12					
		Female	9	10					
Askarifar et al. 2016 [29]	Iran, 6 months RCT	Age, Mean (range)	39.8 (21–62)	43 (19–66)	HL, Non-HL, MM	auto-HCT	CEAM	The ice cubes placed into the patients’ mouth for 5 min before, during and after each chemotherapy dose. To keep it as long as the patient can tolerate it and a maximum of 20 min break allowance	Saline Rinse
		Male	8	9					
		Female	5	7					
Marchesi et al. 2016 [30]	Italy, 2013–2016, RCT	Age, median (range)	58 (42–69)	56 (38–72)	MM	auto-HCT	Melphalan	To place ice chips into the mouth for 15–30 min before starting chemotherapy treatment and continue with the procedure for 6 h following the completion of infusion	No treatment
		Male	26	20					
		Female	10	16					
Kamsvag et al. 2020 [32]	Sweden, 2012- 2016 RCT	Age, < 7 years ≥ 7 years	4 (22)	7 (16)	ALL, AML, LH, SAA, FA, SCD CML, EWS, DS, Lymphoma, MS Neuroblastoma, Thalassemia,	auto-HCT, allo-HCT	Busulfan, Fludarabine, Melphalan, Others	To place ice chips into the mouth during chemotherapy infusion with an intended time of ≥ 30 min	Routine oral care
		Male	15	11					
		Female	11	12					
Lu et al. 2020 [31]	China, 2017–2018, RCT	Age, median (range)	34 (19–62)	35.7 (19–54)	Leukaemia, Lymphoma, MDS, AA	allo-HCT	Busulfan, Cyclophosphamide	Arm A: cryotherapy from the beginning of conditioning regimen infusion until the end. Arm B: From the midpoint of conditioning regimen infusion until the end. Arm C: included as part of daily nursing practice: ice cubes applied for fifteen minutes twice a day during the period of conditioning	Arm D: usual oral care
		Male	26	20					
		Female	12	16					

*RCTs* randomized controlled trials, *OC* oral cryotherapy, *OM* oral mucositis, *TPN* total parenteral nutrition, *auto-HCT* autologous hematopoietic cell transplantation, *allo-HCT* allogeneic hematopoietic cell transplantation, *HL* Hodgkin lymphoma, *Non-HL* non-Hodgkin lymphoma, *MM* multiple myeloma, *ALL* acute lymphoblastic leukaemia, *AML* acute myeloblastic leukaemia, *CML* chronic myeloid leukaemia, *CLL* chronic lymphocytic leukaemia, *HLH* hemophagocytic lymphohistiocytosis, *SAA* severe aplastic anaemia, *AA* aplastic anaemia, *FA* fanconi anaemia, *MDS* myelodysplastic syndrome, *SCD* sickle cell disease, *MS* multiple sclerosis, *EWS* Ewing’s sarcoma, *TC* testicular cancer, *CEAM* conditioning regimen including lomustine, etoposide, cytarabine, and melphalan, *BEAC* conditioning regimen including carmustine, etoposide, cytarabine, and cyclophosphamide, *BEAM* conditioning regimen including carmustine, etoposide, cytarabine, and melphalan

**Table 2:** Characteristics and outcome of included studies

Author	Pain and Mucositis Assessment Tool	Mucositis Status (Grade 3–4)				Study Outcome
Lilleby et al. 2006 [25]	NRS NCI-CTC		Treatment	Control	*P* value	OC was significantly more effective than normal saline mouthwash in reducing the incidence of developing grade 3–4 OM. It also reduces the use of narcotics (*P* = 0.0003) and TPN (*P* = 0.04). The intervention did not reduce length of hospitalization; however, it improves patient's wellbeing
			14%	74%	0.0005	
Gori et al. 2007 [33]	NA WHO-OTS		47%	53%	0.46	In patients receiving low dose Methotrexate chemotherapy, OC did not reduce the incidence of developing sever OM grade3-4
Svanberg et al. 2007 [26], Svanberg et al. 2010 [27]	VAS Modified version of (OMAS)	Day 10^a^	1.60 ± 1.9	4.30 ± 5.7	0.042	OC was significantly more effective than the basic oral care in reducing the incidence of OM grade 3–4. The Use of pain killers, TPN, and duration of hospital stay was also reduced
		Day 16^b^	3.70 ± 1.8	11.6 ± 6.8	0.021	
		2010^c^	23%	52%	< 0.05	
Salvador et al. 2012 [28]	VAS WHO-OTS	Day 6	0.09 ± 0.12	0.05 ± 0.12	0.02	OC was significantly more effective than the usual oral care. However, it did not reduce the duration of hospital stay (*P* = 0.17) or improves nutritional status
		Day 9	0.43 ± 0.12	1.14 ± 0.12	< 0.001	
		Day 12	0.04 ± 0.12	0.41 ± 0.12	0.03	
Askarifar et al. 2016 [29]	NA WHO-OTS	Day 3	0.31 ± 0.17	0.77 ± 0.43	0.112	OC was significantly more effective than normal saline mouthwash in reducing the severity of OC in day 7th and 14th
		Day 7	1.81 ± 0.83	2.54 ± 0.87	0.031	
		Day 14	0.13 ± 0.08	0.92 ± 0.08	0.004	
Marchesi et al. 2016 [30]	NRS NCI-CTC		5.6%	44.4%	0.0002	OC group presented a significant lower occurrence of both grade 3–4 OM but also reduce the need for opioids IV therapy (*p* = 0.001) and TPN use (*P* = 0.005)
Kamsvag et al. 2020 [32]	Children Institutional Oral Mucositis Evaluation Scale FPS (4–6 years old) NRS (≥ 7 years old) WHO-OTS		58%	48%	0.43	OC did not reduce the incidence of sever OM and use of pain killers (*P* = 0.34) and TPN (*P* = 0.35)
Lu et al. 2020 [31]	NA NCI-CTC		24%	39%	0.012	Both arm A and B were associated with a reduction on the incidence and duration of OM compared to arm D

^a^ Autologous, Svanberg et al. 2007 [26], ^b^ Allogeneic/URD, Svanberg et al. 2007 [26], ^c^ Svanberg et al. 2010[27]

The OC incidence rate was reported as percentage (%) in Lilleby et al. 2006 [25], Gori et al. 2007 [33], Svanberg et al. 2010 [27], Marchesi et al. 2016 [30], Kamsvag et al. 2020 [32], and Lu et al. 2020 [31] while in Svanberg et al. 2010 [27], Salvador et al. 2012 [28], Askarifar et al. 2016 [29], as mean ± SD

*WHO-OTS* world health organization-oral toxicity scale, *NCI-CTC* national cancer institute-common toxicity criteria, *OMAS* oral mucositis assessment score, *VAS* Visual Analogue Scale, *NRS* Numerical Rating Scale, *FPS* Face Pain Scale, *OC* oral cryotherapy, *OM* oral mucositis, *TPN* total parenteral nutrition, *NA* not applicable

### Characteristics of the included studies

This review includes all the existing studies on four electronic databases: PubMed, Scopus, Embase and MEDLINE via OvidSP (1946–present), the evidence was current up to 20 October 2020. The studies selected consisted of 9 RCTs published from 2006 to 2020 for both adults and paediatric population in which a total 658 participants were randomised into the experiment group who received OC versus the control group who received no treatment or different interventions, including: basic oral care or saline mouth rinse. It is important to note that only one study in this review utilised mixed interventions approach and found that OC plus oral care consisting of sodium bicarbonate mouthwash reduce the incidence of OM in experimental group compared to OC combined with non-surgical interventions such as low-level laser therapy (LLLT) [28]. Regarding the study design, this review only included RCTs, with eight of these studies having two arms, whilst one study had four arms as summarised in Tables 1  and 2.

In terms of types of interventions the remaining studies had covered, a total of 2 RCTs had investigated the effectiveness of OC versus saline rinse [25, 29], while other 2 RCTs had compared OC with no treatment [30, 33]. Furthermore, studies by Svanberg et al. 2007 [26], Svanberg et al. 2010 [27], Kamsvag et al. 2020 [32] had compared OC to usual or basic oral care. It was essential to highlight that Svanberg and colleagues had conducted two RCTs, the first one in 2007 investigating the efficacy of OC in reducing the incidence of OM and opioid use, while the latter was in 2010 and investigated the efficacy of OC on enhancing nutritional status and reducing hospital stay duration [26, 27]. A recent study by Lu et al. 2020 [31] had taken a broad approach to investigate the efficacy of different duration of OC during the period of conditioning chemotherapy, thus; ice cubes applied from the beginning, from the midpoint and for fifteen minutes twice a day compared to basic oral care.

### Critical appraisal of the included studies

Cochrane tool modified by Higgins et al. 2011 [24] was used for assessing potential sources of bias for this systematic review. The tool consisted of five main domains to be assessed including: selection, performance, detection, attrition and reporting biases. For each domain, the source of bias was evaluated based on the severity of risk: ‘low risk’, ‘high risk’, or ‘unclear risk’ [24]. In terms of the randomisation domain, consisting of two aspects (random sequence generation and allocation concealment), only four studies had provided an adequate description of how the process of randomization was secured and conducted [26–28, 30]. Concerning the blinding of participant and personnel, it was not applicable due to the different physical appearance of the type of interventions, however; RCTs conducted by Salvador et al. 2012 [28] and Kamsvag et al. 2020 [32], had taken action to overcome detection bias by blinding the outcome assessor. Risk of attribution bias was observed in three RCTs due to lack of adequate description of how the missing data (e.g. patients dropped from the study) were treated in the outcome of the statistical analysis [25, 29, 31]. All potential risks of bias are summarized in Table 3.

**Table 3:** Critical Appraisal for RCTs

Author/Year of publication	Random Sequence generation (Selection Bias)	Allocation Concealment (Selection Bias)	Blinding Participant/Personnel (Performance Bias)	Blinding Outcome Assessment (Detection Bias)	Incomplete Outcome Data (Attrition Bias)	Selective Reporting (Reporting Bias)	Other Biases
**Lilleby et al. 2006 **[25]							
**Gori et al. 2007 **[33]							
**Svanberg et al. 2007 **[26]**, Svanberg et al. 2010 **[27]							
**Salvador et al. 2012 **[28]							
**Askarifar et al. 2016 **[29]							
**Marchesi et al. 2016 **[30]							
**Kamsvag et al. 2020 **[32]							
**Lu et al. 2020 **[31]							

The interventional RCTs are listed and identified by author and year of publication. The Cochrane collaboration’s tool for assessing risk of bias in RCTs adopted from Higgins et al. 2011 [24], was used as a critical appraisal tool to identify and highlight potential areas of bias in each included study

Key:  Low risk bias;  High risk of bias;  Unclear risk of bias

### Chemotherapy agents

The alkylating agent, melphalan, used in 6 studies; Lilleby et al. 2006 [25], Svanberg et al. 2007 [26], Svanberg et al. 2010 [27], Salvador et al. 2012 [28], Marchesi et al. 2016 [30] and Kamsvag et al. 2020 [32], was the drug of choice for multiple myeloma (MM) conditioning regimen prior to HCT because it is known to be successful in destroying both dividing and non-dividing tumours cells [34]. Melphalan has been used as a single agent for auto-HCT in MM patients; Lilleby et al. 2006 [25], Salvador et al. 2012 [28], Marchesi et al. 2016 [30] or in combination with busulfan for patients with acute lymphoblastic leukaemia (ALL), acute myeloid leukaemia (AML), chronic myeloid leukaemia (CML), hemophagocytic lymph histiocytosis, severe aplastic anaemia and sickle cell disease, undergoing auto-HCT or allo-HCT [32]. In Svanberg et al. 2007 [26], Svanberg et al. 2010 [27], melphalan was successfully combined with carmustine, etoposide and cytarabine as conditioning regimen for MM, AML, ALL, lymphoma patients, while with lomustine, etoposide and cytarabine in Askarifar et al. 2016 [29] for Hodgkin lymphoma, non-Hodgkin lymphoma, and multiple myeloma patients undergoing auto-HCT and allo-HCT. On the other hand, when the alkylating agent busulfan was used in Kamsvag et al. 2020 [32] and Lu et al. 2020 [31] studies, it had profound toxic effect on non-dividing marrow cells including early myeloid precursors but also lethal to varieties of malignancies such as CML, AML,MM, ALL and lymphomas.

The alkylating antineoplastic agent, cyclophosphamide which was used for Burkitt's lymphoma, acute AML, Hodgkin's and non-Hodgkin's lymphoma, CLL, CML, ALL, T-cell lymphoma (mycosis fungoides), MM and conditioning regimens for HCT[34], was combined with busulfan in [31] as conditioning regimen for leukaemia, lymphoma, aplastic anaemia, and myelodysplastic syndrome patients for HCT, but in Svanberg et al. 2007 [26], Svanberg et al. 2010 [27], it was combined with carmustine, etoposide, and cytarabine for auto-HCT and allo-HCT patients with MM, AML, ALL and lymphoma patients. All these drugs caused different stages of mucositis starting with mouth ulcers and in some cases mouth infections and gastro-intestinal mucositis developed.

### Incidence of oral mucositis

The main objective of all selected studies was to explore the use of OC for management of OM, comparing it to no treatment, oral care or normal saline mouthwash. Three different scoring systems; WHO-OTS [35], NCI-CTC [36] and a modified version of Oral Mucositis Assessment Score (OMAS) [37], were used in these trials, to assess the severity of OM. WHO-OTS was used in 4 trials and NCI-CTC used in 3 studies, with a score of > 3 considered as severe OM in both scoring systems. Severe OM was defined by painful erythema, mouth ulcer, and difficulty in swallowing leading to the need to initiate intravenous hydration.

In Gori et al. 2007 [33], where methotrexate was used to prevent Graft-Versus-Host Disease (GvHD), no significant difference was observed for OM between control and treatment groups estimated by *p* value = 0.46. Similar results were observed for the Kamsvag et al. 2020 [32] study, among children treated with melphalan or busulfan as part of the conditioning regimen, while children who received fludarabine as part of the conditioning regimen showed a lower grade of severe OM (*p* value = 0.34). In two studies, Askarifar et al. 2016 [29] and Salvador et al. 2012 [28], OM was monitored up to 12 and 14 days respectively after infusion of chemotherapeutic agents, and a significant difference was observed in control vs treatment groups. In Salvador et al. 2012 [28], where a single chemotherapy regimen, melphalan was used, the significant differences in OM scores started on day 6 (*p* value = 0.02), peaked on day 9 (*p* value < 0.001), and remained on day 12 (*p* value = 0.03), while in Askarifar et al. 2016 [29], where conditioning regimen consisted of lomustine, etoposide, cytarabine, and melphalan (CEAM), the significant difference was observed on day 7 (*p* value = 0.031) in the OC group compared to the normal saline mouthwash group.

In the studies performed by Svanberg et al. 2007 [26] and Svanberg et al. 2010 [27], where a modified version of OMAS was used to define OM status, the treatment group had a significantly lower mucositis score on day 10 (*p* value = 0.042) compared to the control group in auto-HCT but for allo-HCT patients, the significant difference was observed on day 16 (*p* value = 0.021).

Three studies Lilleby et al. 2006 [25], Lu et al. 2020 [31], and Marchesi et al. 2016 [30] where NCI-CTC system was used to assess OM in control and treatment groups, had presented a significantly lower occurrence of grade 3–4 OM (*p* value < 0.001) among myeloma patients receiving melphalan and OC compared with routine oral care or normal saline mouthwash. In Lu et al. 2020 [31], with a combination chemotherapy (busulfan and cyclophosphamide), the mucositis rate was similar between arm A (*n* = 38, cryotherapy from the beginning of conditioning regimen infusion until the end) and arm B (*n* = 36, from the midpoint of conditioning regimen infusion until the end), and between arm C (*n* = 36, included as part of daily nursing practice: ice cubes applied for fifteen minutes twice a day during the period of conditioning) and arm D (*n* = 35, received oral routine care). The patients in arms A and B treated with cryotherapy, presented a lower incidence of OM compared with those on routine oral care over the observation period. There was no significant difference between arm A and B (*p* value = 0.463), however; both arms showed significant difference from arm D (*p* value = 0.011 and *p* value = 0.068, respectively), while arm C showed no significant difference (*p* value = 0.848) from control group (arm D). The patients in arm C (where ice cubes were applied for fifteen minutes twice a day during the period of conditioning), exhibited more severe mucositis than arm A (*p* = 0.006) and B (*p* value = 0.041) [31].

### Management of oral mucositis

#### Oral care

In four trials, the control group had no special treatment, only a standard procedure was followed for oral care during HCT. The Standard oral care protocol involved check-up of the oral cavity and necessary dental treatment provided by a hospital before the start of the conditioning regimens, followed by oral inspection by nursing staff at the ward [26, 27, 30, 32, 33]. In one trial, Lu et al. 2020 [31] chlorhexidine mouthwash for 3 min, was advised half an hour before and after eating or half an hour before sleeping, while in Salvador et al. 2012 [28] sodium bicarbonate mouthwash was included as part of normal oral care procedure.

#### Normal saline rinse

In two studies, Askarifar et al. 2016 [29] and Lilleby et al. 2006 [25], the control group receiving high-dose conditioning regimens, used normal saline as mouthwash before, during and after chemotherapy. In Lilleby et al. 2006 [25], patients randomised to saline rinses were instructed to use 30 ml of normal saline in the mouth and spit it out every 30 min, while in Askarifar et al. 2016 [29],30–50 ml of normal saline was used for 30 min, before the start of chemotherapy, and every half-hour until six hours after the completion of the course.

#### Oral cryotherapy

In all RCTs, the OC group was instructed to cool their mouths by sucking on ice chips and ice popsicles or rinsing their mouths with ice cold water, during chemotherapy infusions given as conditioning regimens. In all cases OC was started before the chemotherapy and continued for at least the first 30 min, then was stopped according to infusion rate and chemotherapeutic agents’ type. For Methotrexate, administrated as an intravenous infusion lasting about five minutes, OC was started one hour before chemotherapy and stopped by the end of infusion [33]. For Melphalan infusion, patients were trained to continue OC practice for 6 h after the end of the 30-min Melphalan infusion [25] or four times a day with > 12-h infusions [32]. In the, Lu et al. 2020 [31] study, two other groups were also included; in one group OC was introduced from the midpoint of conditioning regimen infusion until the end and in the second group the ice cubes were applied for fifteen minutes twice a day during the period of conditioning.

### Potential benefits of oral cryotherapy

#### Pain management and use of TPN

In terms of the impact of OC on reducing the severity of pain that was associated with chemotherapy-induced OM and enhancing patients’ nutritional status among adult population, only three RCTs addressed these factors clearly, and concluded that the OC groups had experienced a noticeable reduction in the consumption of analgesic drugs and use of total parenteral nutrition (TPN). These RCTs conducted by Marchesi et al. 2016 [30], Svanberg et al. 2010 [27] and Lilleby et al. 2006 [25] had also reported statistically significant decrease on the consumption of narcotics and TPN (Table 2). However, although Salvador and his colleagues reported a significant decrease in the consumption of narcotics drugs among patients receiving OC, there was no evidence of improvement on the nutrition status that was associated with reduction of using TPN [28]. On the other hand, Kamsvag et al. 2020 [32] did not observe any reduction on the use of narcotics and TPN among children population, which could be due to non-compliance with the duration of OC intervention by the young age group participants. In terms of enhancing patients’ activities and quality of life, Lilleby et al. 2006 [25] observed a significant improvement on the activities of swallowing, eating, drinking, talking. Also, patients had reported that taste was less impaired and, moreover, there was an improvement in the pattern of sleeping in the OC group.

#### Reduction of hospital stay

Three studies had agreed that the intervention of OC did not play a significant role in terms of reducing the length of hospitalisation [25, 28, 30]. However, Svanberg et al. 2010 [27] observed a reduction on the duration of hospital stay among the OC group.

## Discussion

Oral mucositis (OM) is one of the adverse effects of chemotherapy that most often exacerbates the overall health of patients receiving HCT with haematological malignancies, in addition to increasing hospitalizations and financial expenses. Different strategies and interventions have been used to minimise the risk of developing OM following high intensity chemotherapy protocol, however the effect of oral cryotherapy (OC) in management of OM is not fully explored. This systematic review reports the benefits of OC in the management of OM produced as an adverse effect of chemotherapy treatment in patients undergoing HCT with haematological malignancies.

Oral mucositis can result from systemic chemotherapy by cytotoxic drugs or radiation to the oral mucosa or the combination of both interventions. It affects approximately 60% to 100% of patients receiving very high doses of chemotherapy before a HCT [38, 39]. Almost all patients receiving combination of both chemotherapy and radiotherapy will develop OM (41). The frequency and severity of mucositis depends on the type, duration and dose of chemotherapy used. The alkylating agents based regimens are recommended in patients undergoing either autologous or allogeneic transplantation, due to their effectiveness in limiting bone marrow toxicity and eradicating dividing and non-dividing tumours cells [6, 34]. Blijlevens et al. 2008 [40] and Castagna et al. 2007 [41] compared the use of melphalan and the BEAM (carmustine, etoposide, cytarabine and melphalan) protocol as conditioning regimens for HCT. Both studies found that approximately 50% of patients developed severe OM associated with both protocols, however it was more prevalent in patients treated with melphalan only. In another study, where two different types of alkylating agents were compared, the degree of OM was 54.4% (24 out of 44 patients) for melphalan and 81.8% (153 out of 187 patients) for busulfan [42]. Although many therapeutic agents have been investigated, there is no effective prevention or treatment standard protocol for management of OM.

The effectiveness of OC was demonstrated in seven studies, where the incidence and severity of OM were found to be significantly (*p* value < 0.05) lower in the treatment group compared to control group, when the conditioning regimen protocols included high dose of alkylating agents such as melphalan, busulfan and cyclophosphamide [25–31]. It is important to consider that melphalan infusion was drug of choice, alone or as a combination therapy, for HCT in six studies because of the short plasma half-life (5 to 15 min) of this drug, allowing the OC procedure to last through the whole infusion session for all patients [25–30, 43]. It has been proposed that reduction in the local temperature leads to vasoconstriction of the oral mucosa and decreases the exposure to melphalan [9, 23]. This temperature-dependent reduction in the cytotoxicity of melphalan also induces impairment in the release of inflammatory chemokines and cytokines that are related to the pathogenesis of OM [44]. OC was also recommended for the management of OM in patients with solid tumours by Mucositis Study Group of the Multinational Association of Supportive Care in Cancer and International Society of Oral Oncology (MASCC/ISOO), in clinical practice, in 2007 and 2013 updates [9, 45]. A systematic review by Correa et al. 2020 [23], also showed that OC was effective in the management of OM in patients undergoing HCT, when melphalan was used as conditioning regimens for HCT. Our present systematic review added two new RCTs; Marchesi et al. 2016 [30] and Lu et al. 2020 [31], which support statements of recommending OC for adult patients undergoing HCT, when conditioning regimen protocols included high dose of alkylating agents such as melphalan, busulfan and cyclophosphamide. While in one RCT with similar family of chemotherapeutic agent; Melphalan, OC did not reduce the incidence of severe OM because the children had greater difficulty in complying with the intervention, since only 58% children used OC as instructed [32]. Considering that in future RCTs, oral cryotherapy can be delivered, using iced water or cubes and flavoured ice popsicle, compliance in children is expected to be good. It is also possible that ice cubes may be a potential choking hazard in children.

Fluorouracil (5FU) is one of the most commonly used drugs to treat solid cancers, in this setting, OC typically involves holding ice chips in the mouth five minutes prior to chemotherapy and continuing for 30 min [10]. Like that of 5-FU, the pharmacokinetics of high-dose melphalan demonstrates a short plasma half-life, which suggest that OC only, during the administration period, could prevent the subsequent OM caused by melphalan. However, the effectiveness of OC during the 30 min of 5-FU chemotherapy infusion is significantly high for management of OM in patients with solid tumours compared to haematology population receiving melphalan [22, 23]. Although both drugs were given as short-term infusions and have short half-life [46] In two studies where OC was started before the infusion of melphalan and was continued for 1 h and 2 h [36, 47], no difference was observed between both groups. A similar observation was made in 2 h compared to 7 h OC treatment in patients receiving melphalan and undergoing autologous HCT [48]. The effectiveness of OC during the 30 min of 5-FU chemotherapy infusion is significantly high for management of OM in patients with solid tumours compared haematology population receiving melphalan [22, 23]. Although both drugs were given as short-term infusions and have short half-life [46], in patients with solid tumours who received 5-FU, OC was more effective [22]. Therefore, an optimal time course for management of OM with OC in HCT with melphalan and other alkylating agents, has yet to be defined.

It has previously been shown that the administration of posttransplant methotrexate for graft-versus-host disease (GvHD) prophylaxis is associated with increased incidence of severe OM following myeloablative allogeneic HCT [49]. OC does not have a significant (*p* value ~ 0.46) role in the prevention of OM in patients receiving methotrexate containing GvHD prophylaxis following HCT [33]. The reasons for this inconsistency, may be related to mode of administration, plasma half-life (3 to 15 h), optimal duration of OC or biological effect of posttransplant low-dose methotrexate in the pathogenesis of oral mucositis. A new drug, palifermin reduces the incidence of mucositis after high dose methotrexate, however, the clinical benefit fell short of expectations and did not reach statistical significance in allogeneic HCT [50].

There were a few limitations in this review; mainly due to heterogeneity of the studies such as diagnosis definition and chemotherapeutic agent regimen. Also, there was variation in terms of reporting the results, for instance, two different Mucositis Assessment Tools (WHO-OTS and NCI-CTC). Moreover, there was a wide variation on duration of the OC interventions, ranging from 15 min [31] to 6 h [25]. The pain measurement scales were an additional limitation: either not reported or Numerical Rating Scale (NRS) and Visual Analogue Scale (VAS) was used in these trials.

In conclusion, our study provided significant data to support that oral cryotherapy is more effective in the management of oral mucositis in patients with haematological malignancies prior to haematopoietic cell transplant, especially if high dose melphalan is given as conditioning protocols. Therefore, considering the lower costs and safer intervention, it is suggested to use oral cryotherapy in the recovery of oral mucositis in these patients. However, the heterogeneity of these trials demonstrated the need to regulate the validated assessment tools and similar interventions that would enable comparisons and analyses of treatment effects based on well-designed randomised controlled trials.

## Data Availability

The datasets used and/or analysed during the current study are available from the corresponding author on reasonable request.

## Abbreviations

ALL: Acute Lymphoblastic Leukaemia
AML: Acute Myeloid Leukaemia
CIOM: Chemotherapy-induced oral mucositis
CML: Chronic Myeloid Leukaemia
HLH: Hemophagocytic Lymph Histiocytosis
HCT: Haematopoietic cell transplant
LLLT: Low-level laser therapy
MM: Multiple myeloma
NRS: Numerical Rating Scale
OM: Oral mucositis
SAA: Severe Aplastic Anaemia
SCD: Sickle Cell Disease
TPN: Total parenteral nutrition
VAS: Visual Analogue Scale
